# Performance characteristics of a light-scattering immunoassay for thyroxine on a discretionary analyser

**DOI:** 10.1155/S146392468700021X

**Published:** 1987

**Authors:** Roy Jaggon, Christopher P. Price

**Affiliations:** Department of Clinical Biochemistry Addenbrooke's Hospital Hills Road Cambridge CB2 2QR UK


Journal of Automatic Chemistry/*Journal of Clinical Laboratory Automation*, Vol. 9, No. 2 (April–June 1987), pp. 97–99

### Performance characteristics of a light-scattering immunoassay for thyroxine on a discretionary analyser

**Roy Jaggon and Christopher P. Price***
*Department of Clinical Biochemistry, Addenbrooke's Hospital, Hills Road, Cambridge CB2 2QR, UK*

### Introduction

The use of changes in light-scattering properties as a means of monitoring the reaction between two species has been used for many years. In recent years interest has tended to concentrate on monitoring the turbidity of a reaction mixture. The reaction between polyvalent antigens and the appropriate antibodies may be monitored by the development of the turbidity as a result of immunoaggregate formation; this technique is used routinely for the quantitation of specific proteins. Reactions are monitored at 340 nm for short periods of time, generally less than 10 min and, as a consequence, results can be produced quickly.

The advent of the discretionary analyser capable of performing many different types of analyses has in general terms favoured the employment of methods with short reaction times to improve the throughput of analyses. The short reaction times demonstrated by immunoturbidimetric techniques favours their use on this type of analyser.

The formation of a light-scattering immunoaggregate requires that the antigen is polyvalent. This technique can, however, be employed for the measurement of a small monovalent antigen; the analyte (hapten) in the sample inhibits the formation of an immunoaggregate formed from the antibody and a polyvalent antigen synthesized from the hapten linked to a large molecule or light-scattering particle.

An assay has been developed for the measurement of thyroxine whereby thyroxine in the patient's sample inhibits the formation of an immunaggregate from antibody bound to latex particles and thyroxine conjugated to Ficoll. Trypsin is included in the reaction mixture to minimize interference from serum proteins. We report our experience of this assay using an RA1000 discretionary analyser (Technicon Instruments, Basingstoke, UK).

### Materials and methods

The Technicon Total Thyroxine reagents were prepared exactly according to the manufacturer's instructions. The system comprises a buffered reagent containing a thyroxine-Ficoll conjugate and trypsin and a reagent containing monoclonal antibodies to thyroxine bound to latex particles.

Briefly, the method involves the addition of sample (26 μl) to the thyroxine-Ficoll conjugate reagent (190 μl). After an incubation of 4 min, 190 μl of the thyroxine antibody reagent is added. After a delay of 1 min the increase in turbidity is measured at 600 nm for a period of 135 s.

A set of six calibrators with assigned thyroxine values can be purchased from Technicon Instruments.

### Experimental procedures and results

*Precision*

Three commercial lyophilized serum preparations were employed in this study (Lyphocheck I, II and III, Biorad, Watford, UK). Fresh bottles of each material were reconstituted at the beginning of each week; the reconstituted material was stored at 4 °C.

Twenty aliquots of each material were run in one batch to establish the within-run precision. The data are shown in table 1.

*Table 1. Imprecision ($N = 20$) of light-scattering thyroxine assay.*

| | Mean thyroxine nmol/l | SD | CV (%) |
|---|---|---|---|
| Within-batch | 23·1 | 1·95 | 8·4 |
| | 103·9 | 2·12 | 2·0 |
| | 201·6 | 3·35 | 1·7 |
| Between-day | | | |
| Daily calibration | 25·2 | 2·19 | 8·7 |
| | 99·5 | 5·15 | 5·2 |
| | 197·2 | 11·07 | 5·6 |
| Weekly calibration | 28·2 | 5·20 | 18·5 |
| | 99·9 | 6·77 | 5·8 |
| | 206·2 | 7·43 | 3·6 |

A calibration curve was established on each day for a period of 20 working days. Each of the reconstituted materials was analysed, once on each working day. The thyroxine level on each day was determined using the calibration curve established on the first day of the week

* *Correspondence to this author.*

and that established on each working day. The data are shown in table 1.

A typical calibration curve is shown in figure 1.

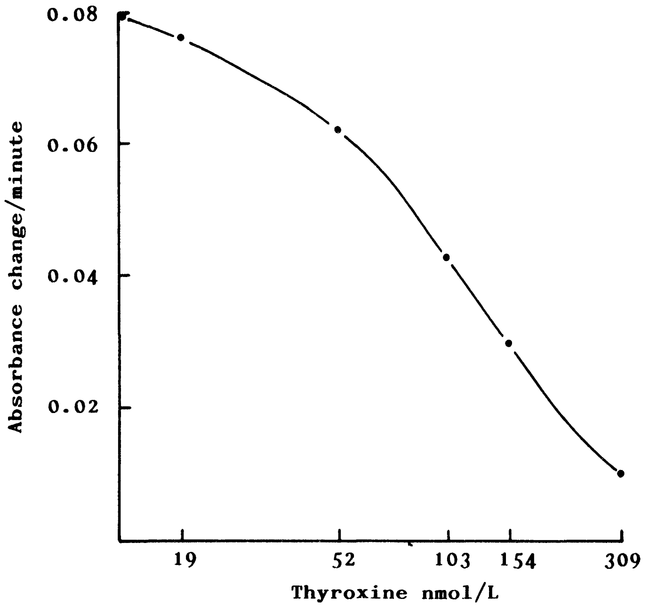

*Figure 1. A typical calibration curve for the light-scattering immunoassay of thyroxine.*

*Linearity*

A sample with a high thyroxine level was diluted with zero calibrator in varying proportions and duplicate determinations of the thyroxine level made. Regression analysis of the data gave a correlation coefficient of 0·9987 when the observed value was compared with the expected value.

*Detection limit*

The zero calibrator was analysed 10 times in one batch and the absorbance change used to determine the mean absorbance change (0·0796 min) and the standard deviation (0·008). The detection limit defined as the signal change equal to three times the standard deviation of the base-line was calculated from the initial calibration curve as 4·1 nmol/l (in the sample).

*Method comparison*

The thyroxine level was determined using the turbidimetric method on 106 patients samples; the thyroxine level was also determined using an in-house radioimmunoassay. The data are shown in figure 2. The radioimmunoassay was calibrated with material obtained from Ciba Corning (MAGIC calibrator set, Ciba Corning, Halstead, UK).

*Recovery of pure analyte*

Thyroxine (obtained from Sigma Chemical Company, Poole, Dorset, UK) was dissolved in ethanol. A one in 500 dilution of this stock was made in a 100 mmol/l phosphate buffer, pH 7·4, containing 10 g/l bovine serum albumin.

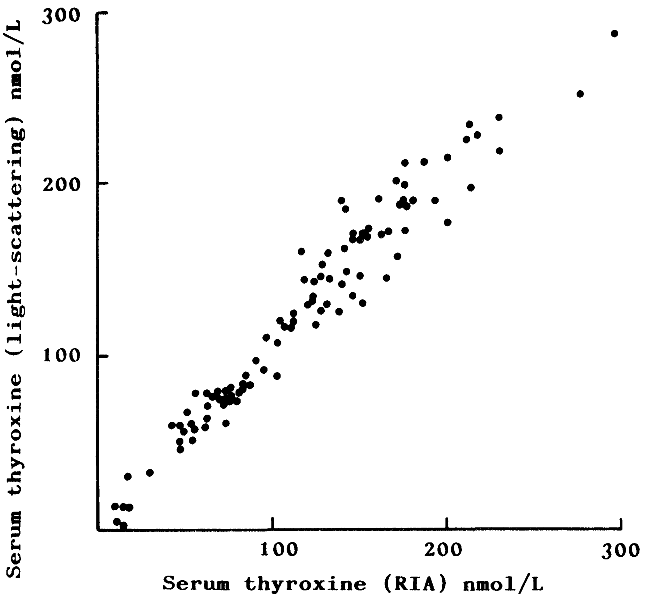

*Figure 2. Comparison of thyroxine results obtained with the light-scattering(y) and radio-immunoassay(x) methods. Regression analysis using the method of Deming [3] gave a correlation coefficient(r) of 0·971 with a line y = 0·976x + 7·37, n = 100.*

This substock had a calculated thyroxine level of 2250 nmol/l. The substock was then diluted one in 41, one in 21 and one in 11 with the zero calibrator and the thyroxine level assayed in duplicate. The mean recovery at a level of 55 and 107 nmol/l was 100·3% (range 96·2–103·4%). At a level of 205 mmol/l the recovery was 117% (range 116·4–118.3%).

Analysis of the Technicon calibrators by radioimmunoassay indicated a level of 318 nmol/l at an assigned value of 308 nmol/l with agreement at the lower levels (less than 3% difference) whilst analysis of the Ciba Corning calibrators in the light-scattering method indicated an underestimation of 13 nmol/l at a level of 193 nmol/l, with agreement at the lower levels.

*Endogenous interferents*

*Haemolysis, icterus, lipaemia:* a serum sample was spiked with an haemolysate and bilirubin solution to varying levels. The thyroxine level of each mixture was then assayed in duplicate. The results indicate the mean effect on the apparent thyroxine level was a decrease of 10 nmol/l in the presence of haemoglobin at a concentration of 0·5 g/dl. There was a gradual increase in the apparent thyroxine level equivalent to 21 nmol/l at a bilirubin level of 500 umol/l. The thyroxine level of six lipaemic serum samples (triglyceride levels in the range 3·9–34·9 mmol/l) were assayed by the light-scattering and radioimmunoassay methods. No significant difference in the results was noted.

*Elevated TBG levels:* a total of 29 serum samples with TBG levels greater than 13 mg/l were assayed for thyroxine by the light-scattering and radioimmunoassay methods. The data shown in figure 3 indicate no correlation between TBG levels and difference in results between the two methods.

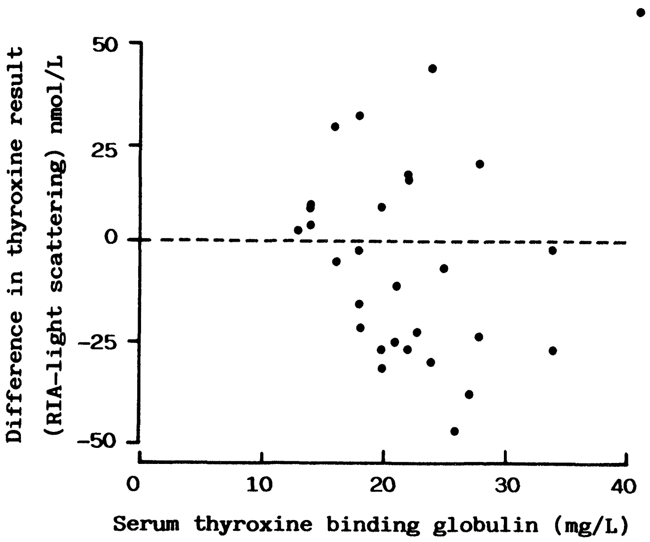

*Figure 3. Difference in thyroxine results obtained by the light-scattering and radio-immunoassay methods in samples with high levels of thyroxine-binding globulin.*

*Paraproteins and autoantibodies:* a total of 16 samples known to contain elevated levels of rheumatoid factor, 35 samples containing paraproteins (concentration range 5-40 g/l), eight samples containing mitochondrial antibodies and six samples containing nuclear factor antibodies were all assayed for thyroxine by the light-scattering and radioimmunoassay methods. The data are shown in figure 4.

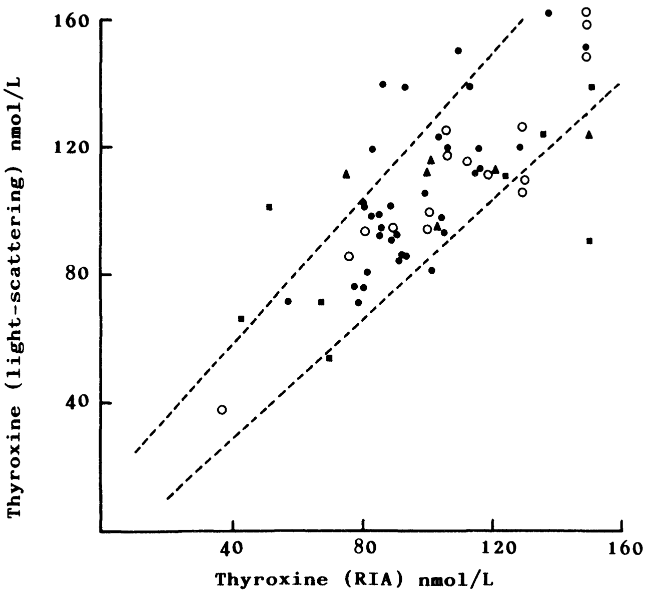

*Figure 4. Comparison of thyroxine results obtained with the light-scattering and radioimmunoassay method with samples containing paraproteins (●) (IgG, IgA or IgM) rheumatoid factor (○) mitochondrial antibodies (■) or nucleur factor antibodies (▲). The dotted lines denote the limits of the results obtained in the main method comparison (see figure 3).*

### Discussion

Immunoassay methods based on aggregate formation, particularly those where polyethylene glycols are added to the reaction mixture, are typically characterized by short reaction times. Turbidimetric immunoassays have been described for a variety of proteins and haptens where reaction times are less than 5 min. This also applies to assays that employ particle enhancement to improve the sensitivity of the method. In the method described calibration can be completed in about 12 min and about 65 patient samples can be analysed in 1 h.

The reagents developed for this light-scattering assay were found to be robust; it was found that calibrating once a week showed no gross deterioration in method precision compared with a daily calibration routine, although the precision at low thyroxine levels was poorer.

The results obtained with the light-scattering immunoassay showed reasonably good agreement with the radioimmunoassay method. The recovery experiments and analysis of calibration materials suggested a discrepancy at higher levels of thyroxine. This may simply reflect the values assigned to the calibration materials.

There appeared to be no consistent interference in the presence of increased levels of thyroxine binding globulin. Analysis of samples containing increased antibody titres did not show the same degree of concordance of results with radioimmunoassay method although no consistent interference was observed.

The method described in this report is capable of adaptation to discrete analysers found in most clinical laboratories. This offers the possibility of more flexibility in laboratory organization as a result of the employment of a non-isotopic, rapid automated immunoassay. This may be of value as clinical biochemists assess the changing role of the thyroxine assay with the advent of the more sensitive thyrotropin assays [4].

### Acknowledgements

We would like to acknowledge the provision of the reagents by Technicon Instruments (Basingstoke, UK). Thanks are also due to colleagues for provision of data from the radioimmunoassay procedure and to Dr P. M. Clark for helpful advice

### References

1. Price, C. P., Spencer, K. and Whicher, J., *Annals of Clinical Biochemistry*, **20** (1983), 1.
2. Galvin, J. P., in *Diagnostic Immunology: Technology Assessment and Quality Assurance* (College of American Pathologists, Skokie, 1983), p. 18.
3. Cornbleet, P. J. and Gochman, N., *Clinical Chemistry*, **2** (1979), 432.
4. Martino, E., Bambini, G., Bartalena, L., Mammoli, C., Aghoni-Lombardi, F., Baschieri, L. and Pinchera, A., *Clinical Endocrinology*, **24** (1986), 141.

